# The Investigation of Preoperative Factors Associated With Postoperative Outcomes Following Total Knee Arthroplasty for Osteoarthritis: A Scoping Review

**DOI:** 10.7759/cureus.64989

**Published:** 2024-07-20

**Authors:** Takafumi Nasu, Junya Yamanoi, Takashi Kitagawa

**Affiliations:** 1 Department of Rehabilitation Medicine, Juko Osu Hospital, Nagoya, JPN; 2 Department of Physical Therapy, Shinshu University, Matsumoto, JPN

**Keywords:** preoperative factors, predictors, discharge destination, total knee arthroplasty (tka), osteoarthritis of the knee

## Abstract

This study aimed to investigate preoperative factors associated with non-home discharges from acute care hospitals in patients undergoing total knee arthroplasty (TKA) due to osteoarthritis. It was a scoping review focused on patients who received their first unilateral TKA for osteoarthritis. The research targeted observational studies that examined the destinations of patients post-surgery based on preoperative factors, with a literature search conducted in April 2023. Out of 3,255 identified papers, 28 met the eligibility criteria. A total of 26 preoperative factors were identified as potentially related to discharge destinations, including age, gender, comorbidities, and obesity. By selecting an appropriate discharge destination based on preoperative factors, there may be potential for more efficient use of medical resources. Future studies should consider preoperative factors in the context of national healthcare systems and lengths of hospital stay.

## Introduction and background

Total knee arthroplasty (TKA) is commonly performed as a treatment for osteoarthritis, which causes pain, decreased physical function, and reduced activities of daily living, and is considered one of the most cost-effective surgeries [1]. Its frequency is on an increasing trend in many developed countries, with projections indicating a 673% increase in the United States by 2030 [2]. In Japan, the number of surgeries exceeded 70,000 in 2021, and it is expected to continue to rise annually until 2030 [3-5]. The rapid increase in TKA patients leads to significant economic burdens on healthcare systems, necessitating reductions in healthcare costs to sustain the current systems [6-7]. The costs associated with TKA are primarily due to hospitalization expenses, and there is a trend toward reducing the length of hospital stays [8].

In the United States, the average length of stay in acute care hospitals has been decreasing annually, reaching two to three days [9]. Regarding discharge destinations, the short duration of hospital stays may contribute to home discharge rates ranging from 10% to 40% [10-11]. In contrast, in Japan, the average length of stay in acute care hospitals is about 24 days [12]. Due to the longer hospital stays compared to Western countries, 80-90% of patients are reported to return home [12], although a certain number traditionally transfer to rehabilitation hospitals. Nagaoka and Nitta have suggested that differences in the medical insurance system and the number of acute care beds per capita influence the length of hospital stays in Japan [13]. It is unclear if the high rate of home discharge can be maintained if hospital stays decrease like in other countries. Japan must also efficiently utilize medical resources and select appropriate discharge destinations in anticipation of the increasing number of TKA patients each year [14]. Considering discharge destinations preoperatively is essential for smooth discharge planning within limited postoperative hospital stays. Prior studies examining preoperative factors related to discharge destinations have indicated that being female is a predictor for discharge to rehabilitation facilities [15]. However, these studies did not include research conducted in Japan. A comprehensive examination of the latest preoperative factors, including studies from Japan, is expected to provide valuable insights for determining appropriate discharge destinations.

Therefore, we comprehensively collected information on preoperative factors associated with the transfer from acute care hospitals to rehabilitation hospitals or discharge destinations other than home after TKA due to osteoarthritis of the knee. We summarized the current evidence from various countries, including Japan.

## Review

Subjects and methods

This scoping review was conducted to examine preoperative factors related to outcomes following TKA for osteoarthritis of the knee. This study was conducted according to the Preferred Reporting Items for Systematic Reviews and Meta-Analyses (PRISMA) extension for Scoping Reviews [16]. Additionally, the research protocol was registered in advance with the Open Science Framework, following the methodology for scoping reviews.

The selection criteria of the study were determined using the Patient, Concept, Context (PCC) framework [17]. The subjects were patients in acute care hospitals who underwent unilateral TKA due to osteoarthritis, with no restrictions on age or gender. Excluded were patients who underwent unicompartmental knee arthroplasty, revision surgeries, bilateral surgeries, or had postoperative mobility restrictions such as non-weight bearing. Although postoperative hospital stay lengths vary internationally due to different insurance systems, this study focused on preoperative factors related to non-home discharge destinations (such as transfers to rehabilitation hospitals or other facilities). Factors unrelated to patients such as hospital functions, size, and regional differences were excluded from the study. The preoperative factors considered included inherent factors such as age and gender, as well as physical functionality, economic factors, and family presence. Observational studies were employed for the included papers. Studies using large databases were also employed. Case reports, interventional studies, systematic reviews, meta-analyses, and narrative reviews were excluded.

Search Method

Database searches were conducted using MEDLINE, Embase, PEDro, and Google Scholar in English. Additionally, to conduct a search for grey literature, OpenGrey was used. To further examine preoperative factors specific to Japan, a hand search of major Japanese journals was performed. The searches in each database were conducted using search formulas based on the terms "total knee arthroplasty," "preoperative factors," "outcomes," "predictive factors," and "preoperative factors." The literature search was conducted on April 1, 2023, and included papers published up to March 2023. (Table 1).

**Table 1:** Search strategy

Search strategy
MEDLINE (via PubMed) search strategy
("Arthroplasty, Replacement, Knee"[mh] OR "Knee Prosthesis"[mh] OR "knee replacement"[tiab] OR "total knee arthroplasty"[tiab] OR "TKR"[tiab] OR "TKA"[tiab] OR "KR"[tiab]) AND ("predict*"[tiab] OR "determin*"[tiab] OR "preoperative"[tiab] OR "factors"[tiab] OR "characteristic*"[tiab] OR "influence"[tiab] OR "affects"[tiab]) AND ("discharge*"[tiab] OR "Patient Discharge"[mh] OR "discharge disposition"[tiab] OR "discharge destination"[tiab] OR "rehabilitation ward"[tiab] OR "inpatient rehabilitation"[tiab] OR "Hospitals, Rehabilitation"[mh] OR "Subacute Care"[mh])
Embase (via Elsevier) search strategy
('Arthroplasty, Replacement, Knee'/exp OR 'Knee Prosthesis'/exp OR 'knee replacement':ti,ab OR 'total knee arthroplasty':ti,ab OR TKR:ti,ab OR TKA:ti,ab OR KR:ti,ab) AND (predict*:ti,ab OR determin*:ti,ab OR preoperative:ti,ab OR factors:ti,ab OR characteristic*:ti,ab OR influence:ti,ab OR affects:ti,ab) AND (discharge*:ti,ab OR 'Patient Discharge'/exp OR 'discharge disposition':ti,ab OR 'discharge destination':ti,ab OR 'rehabilitation ward':ti,ab OR 'inpatient rehabilitation':ti,ab OR 'Hospitals, Rehabilitation'/exp OR 'Subacute Care'/exp)

Article Selection Method

After completing the database searches, results were uploaded to Rayyan, and duplicates were removed. Two authors (TN, JY) independently selected articles, consulting another author when opinions diverged. Initially, about 10 articles were excluded based on titles and abstracts during a pilot screening using pre-established eligibility criteria. For articles whose full texts were not accessible, authors were contacted via ResearchGate to obtain the documents. Subsequently, a secondary screening was conducted to verify full texts, and only those meeting the eligibility criteria were included in the study.

Data Extraction

The items for data extraction were developed based on the PCC framework. Two authors (TN, JY) independently conducted data extraction from the eligible articles. Disagreements were resolved through discussion, and if no agreement could be reached, a third author was consulted to resolve the issue. Data extracted included the objectives of the studies, demographic information (age, gender) of the participants, discharge destinations, average length of stay, and preoperative factors related to outcomes.

Integration of Results

A PRISMA flowchart was created to summarize the search results and literature selection. The extracted data were organized into tables using Excel, presenting information on age, gender, number of subjects, length of hospital stay, and preoperative factors.

Results

Study Selection

**Figure 1:**
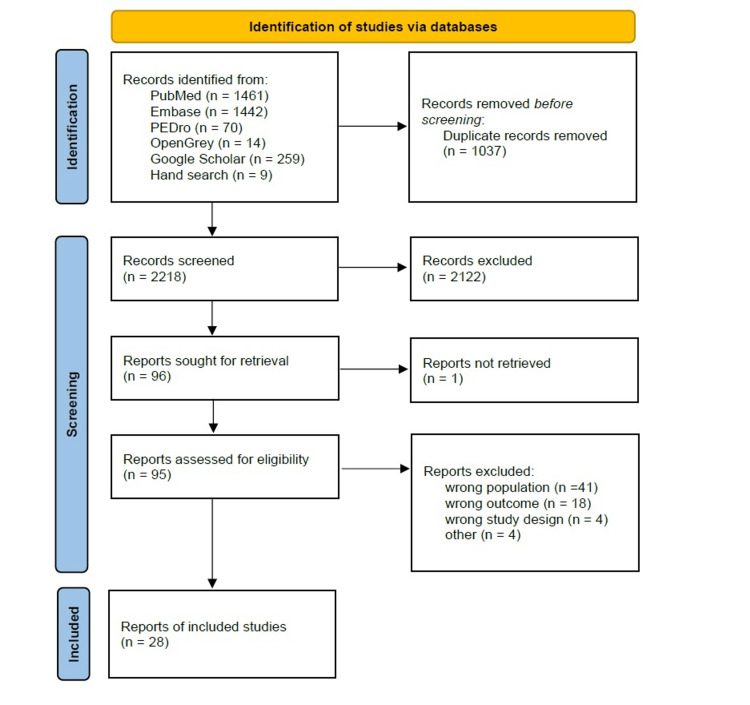
PRISMA flowchart for paper selection PRISMA: Preferred Reporting Items for Systematic Reviews and Meta-Analyses

From the database search, 3,255 articles were identified, and ultimately 28 met the eligibility criteria and were included [18-45]. A flowchart is shown in Figure 1. The selected studies involved observational research on patients undergoing TKA due to osteoarthritis, focusing on preoperative factors and discharge destinations. Studies ranged from single-site investigations to those utilizing large-scale databases. Of the selected articles, 23 were from the United States, 3 from Japan, 1 from Singapore, and 1 from Canada.

Characteristics of Selected Studies

The studies selected examined preoperative factors related to non-home discharge destinations. Most studies targeted older adults, with an average age over 65, and tended to involve more female participants. Reports from the United States indicated nearly all patients were discharged within five days post-operation, identifying 26 preoperative factors, including patient-specific and environmental factors (Tables 2-3).

**Table 2:** Characteristics of the studies and subjects covered USA: the United States of America; SD: standard deviation; SNF: skilled nursing facility; CH: community hospitals; HCV-C: hepatitis C virus was cured; HCV-UT: hepatitis C virus was untreated; MetS: metabolic syndrome

Author and year of publication	Country of origin	Age (years)	Gender (female)	Sample size	Length of stay (days)
Neuwirth et al. 2022 [18]	USA	60>: 23.1%, 60-70: 42.0%, 71-80: 27.3%, 81≦: 7.6%	62%	1,98,233	NA
Issa et al. 2021 [19]	USA	Mean; 68 (41-87)	72.50%	229	3 (1-22)
Zeng et al. 2021 [20]	USA	Mean; 67.6 (SD 8.6)	60.10%	1,481	NA
Gwam et al. 2020 [21]	USA	Mean; home: 66 (SD 9.1), SNF: 71 (SD 9.3), rehabilitation 69 (SD 9.7)	Home: 59.1%, SNF: 72.8%, rehabilitation: 69.5%	1,71,093	NA
Rondon et al. 2017 [22]	USA	Mean; home: 65.7, rehabilitation: 73.8	Home: 57.3%, rehabilitation: 79.4%	2,281	Home: 2.0, rehabilitation: 3.4
McLawhorn et al. 2017 [23]	USA	65≧: 45.1%, 66-75: 35.2%, 76≦: 19.7%	62.40%	1,01,256	NA
Chan et al. 2018 [24]	Singapore	Mean; home: 67.2 (SD 7.8), CH: 70.7 (SD 7.7)	Home: 77.5%, CH: 86.7%	1,065	Home: NA, CH: 23 (17-32)
Black et al. 2019 [25]	USA	NA	NA	2,058	Albumin≦3.5 g/dL: 3.2, albumin>3.5 g/dL: 2.3
Prohaska et al. 2017 [26]	USA	Mean; 63.4 (SD 11.3)	59%	679	NA
Robinson et al. 2017 [27]	USA	Mean; male: 66.5 (SD 9.67), female: 66.8 (SD 9.82)	62.30%	87,177	Hospitalization for more than 3 days, male 7.4%, female 7.9%
Rissman et al. 2015 [28]	USA	Mean; 64.7 (SD 10.4)	57%	738	3.3 (1.2-13.5)
Lung et al. 2023 [29]	USA	Mean; dehydration level none: 65.4, moderate: 67.6, severe: 68.5	Dehydration level none: 45.5%, moderate: 31.8%, severe: 19.3%	3,44,744	Severe dehydration is likely to keep the patient in the hospital for more than two days
Hadad et al. 2022 [30]	USA	Mean: black 64; white 67	Black: 55%, white: 45%	3,603	Blacks are more likely to stay in the hospital for more than 3 days
Schwarzkopf et al. 2019 [31]	USA	Mean; HCV-C: 63.0 (SD 7.5), HCV-UT: 61.7 (SD 6.9)	HCV-C: 46.9%, HCV-UT: 46.1%	127	HCV-C: 2.9, HCV-UT: 3.4
Kerbel et al. 2021 [32]	USA	Mean; 65.1 (17.9-87.7)	66.20%	1,614	Non-obese (<30 kg/m2): 2.6 (SD 1.3), obese (30-39 kg/m2): 2.5 (SD 1.0), morbidly obese (≧40 kg/m2): 2.8 (SD 2.0)
Schwarzkopf et al. 2016 [33]	USA	Mean; 68.7	62.70%	28,611	NA
Chen et al. 2023 [34]	USA	Mean; national cohort 67 (58-76), institutional cohort 69 (60-78)	National: 59.5%, institutional: 62.8%	National: 424,354, institutional: 10,196	National: 2.3 (SD 1.9), institutional: 2.8 (SD 1.4)
Ramkumar et al. 2019 [35]	USA	Median; SNF: 72 (SD 9.7), non-SNF: 65 (SD 10.1)	SNF: 72.3%, non-SNF: 61.2%	32,18,419	SNF: 3.7 (SD 1.8), non-SNF: 3.1 (SD 1.6)
Pan et al. 2023 [36]	USA	Mean; home: 66.1 (SD 9.2), non-home: 70.6 (SD 9.7)	Home: 59.3%, non-home: 70.7%	4,62,858	2011 years: home: 3.3 (SD 4.5), non-home: 3.7 (SD 5.9), 2020 years: home: 1.4 (SD 1.6), non-home: 3.6 (SD 3.1)
Jorgenson et al. 2015 [37]	USA	65>: 56,575, 65≦: 72,947	64.20%	1,29,522	NA
Krishnan et al. 2021 [38]	USA	Mean; cognitive: 75.75 (SD 6.27), non-cognitive: 75.32 (SD 6.17)	Cognitive: 69.2%, non-cognitive: 70.1%	208	Cognitive: 3.62, non-cognitive: 3.35
Shichman et al. 2023 [39]	USA	Mean; MetS: 64 (56-72), No MetS: 64 (55-73)	MetS: 76.5%, No MetS: 77%	782	MetS: 3.4 (1-5.8), No MetS: 3.0 (1.6-4.5)
Kooner et al. 2021 [40]	Canada	Mean; psychiatric: 66.7 (SD 9.7), non-psychiatric: 66.7 (SD 9.6)	Psychiatric: 62.8%, non-psychiatric: 62.8%	1,000	Psychiatric: 6.0 (SD 7.00), non-psychiatric: 5.23 (SD 6.18)
Rudy et al. 2020 [41]	USA	55>: 10.7%, 55-64: 26.9%, 65-74: 39.2%, 75≦: 23.2%	63.30%	1,910	3.2 (2.3-3.3)
Crawford et al. 2011 [42]	USA	Mean; 63.97 (SD 9.90)	51.80%	413	4.35
Takada et al. 2021 [43]	Japan	Mean; home: 71.4 (SD 8.9), hospital transfer: 76.1 (SD 8.1)	Home: 62%, hospital transfer: 88%	89	Median home: 18, hospital transfer: 17
Oki et al. 2020 [44]	Japan	Mean; 71.8 (SD 10)	NA	60	NA
Yamada et al. 2019 [45]	Japan	Mean; home: 69.2 (SD 9.9), hospital transfer: 77.7 (SD 7.5)	71%	72	Hospitalization for more than 14 days

**Table 3:** Preoperative factors associated with the included studies and outcome destinations other than home BMI: body mass index; CCI: Charlson Comorbidity Index; ASA-AP: American Society of Anesthesiologists Physical Status; TUG test: timed up and go test

Author	Country of origin	Preoperative factors
Neuwirth et al. 2022 [18]	USA	Preoperative anemia, hematocrit ≥ 36%, hemoglobin > 12 g/dl
Issa et al. 2021 [19]	USA	Age (older)
Zeng et al. 2021 [20]	USA	Age (older), use of walking aids, exercise habits, dementia, neurological disorder
Gwam et al. 2020 [21]	USA	Age (older), gender (female), race (Black), diabetes, BMI (high), preoperative white blood cells, hematocrit (low)
Rondon et al. 2017 [22]	USA	Age (older), gender (female), insurance status (Medicare), race (non-Caucasian), comorbidities (CCI), history of depression
McLawhorn et al. 2017 [23]	USA	Age (older), gender (female), BMI (high), comorbidities (CCI, ASA-AP), preoperative functional status (non-independent)
Chan et al. 2018 [24]	Singapore	Age (older), gender (female), lower education
Black et al. 2019 [25]	USA	Low albumin level (<3.5 g/dl)
Prohaska et al. 2017 [26]	USA	Age (older), gender (female), preoperative anemia, preoperative obesity
Robinson et al. 2017 [27]	USA	Gender (female)
Rissman et al. 2015 [28]	USA	Age (older), gender (female), comorbidities (CCI), preoperative obesity, preoperative pain catastrophizing scale
Lung et al. 2023 [29]	USA	Dehydration (moderate to severe)
Hadad et al. 2022 [30]	USA	Race (Black)
Schwarzkopf et al. 2019 [31]	USA	Untreated hepatitis C virus, insurance status
Kerbel et al. 2021 [32]	USA	Obesity (30-39 kg/m²), morbidly obese (≥ 40 kg/m²)
Schwarzkopf et al. 2016 [33]	USA	Age (older), comorbidities (CCI), race (Asian, Black), insurance status
Chen et al. 2023 [34]	USA	Age (>68), gender (female), BMI (>33.35 kg/m²), comorbidities
Ramkumar et al. 2019 [35]	USA	Age (older), gender (female), race (Black), comorbidities (CCI)
Pan et al. 2023 [36]	USA	Age (older), gender (female), obesity (BMI > 40 kg/m²), comorbidities (ASA-AP, CCI), race (Black, Asian), preoperative functional status (non-independent), smoker
Jorgenson et al. 2015 [37]	USA	Race (African American)
Krishnan et al. 2021 [38]	USA	Preoperative cognitive impairment
Shichman et al. 2023 [39]	USA	Morbidly obese, metabolic syndrome
Kooner et al. 2021 [40]	Canada	Psychiatric illness
Rudy et al. 2020 [41]	USA	Married/partnered, comorbidities (CCI), insurance status
Crawford et al. 2011 [42]	USA	Age (older), comorbidities (ASA-AP)
Takada et al. 2021 [43]	Japan	Non-operative side extensor strength
Oki et al. 2020 [44]	Japan	TUG test, comfortable gait speed
Yamada et al. 2019 [45]	Japan	Comfortable gait speed

Primary Characteristics (Preoperative Factors)

Among the 26 identified factors, 9 were frequently reported across multiple studies.

Age: Thirteen studies identified age as a factor influencing non-home discharges. Older patients were more likely to be discharged to destinations other than home compared to those who were discharged home. Studies categorizing age showed an increased likelihood of non-home discharges with increasing age.

Gender: Ten studies found that females were more likely than males to be discharged to non-home destinations.

Comorbidities: Nine studies cited comorbidities as influencing discharge destinations, using indices like the Charlson Comorbidity Index (CCI) and the American Society of Anesthesiologists Physical Status (ASA-PS). Higher scores and classifications on these indices were associated with an increased likelihood of non-home discharges.

Obesity: Body mass index (BMI) over 30 kg/m^2^ was associated with an increased likelihood of non-home discharges in nine studies, with even higher probabilities for BMIs over 40 kg/m^2^.

Other: Other factors identified in at least two studies that increased the likelihood of non-home discharges included race (Black), insurance type (Medicare), anemia, low preoperative functional independence, and cognitive impairment.

Discussion

The study showed that preoperative factors such as age, gender, comorbidities (CCI, ASA-PS), and obesity are associated with non-home discharge destinations after TKA. From the selected articles, 26 preoperative factors were extracted, with nine being commonly reported in at least two articles. Moreover, it was evident that not just one, but multiple preoperative factors are interconnected and influence the discharge destination. The selected studies were predominantly from the United States, where nearly all patients were discharged within five days. In contrast, Japanese studies reported hospital stays exceeding two weeks, highlighting significant differences in length of stay. When comparing preoperative factors, U.S. studies frequently selected patient-specific factors, whereas Japanese studies extracted factors related to physical function and performance, such as lower limb strength and walking speed. This indicates the need to adapt preoperative factors considering the healthcare systems and hospital stay durations specific to each country or region.

Age has been reported in prior studies to influence longer hospital stays and transfers [46-47], and this study supports these findings with age being highlighted in 13 papers. Generally, as age increases, so does the number of comorbidities [48], which can prolong the time needed for postoperative functional recovery [49]. Additionally, reports suggest that females may require more time to regain daily living activities, extending their hospital stays [46]. The most common comorbidities in TKA patients are reported to be hypertension (67.74%), obesity (21.73%), and diabetes (19.83%), indicating that many TKA patients have multiple comorbidities [50]. These patient-specific factors - age, gender, and comorbidities - are commonly selected for these reasons. However, while obesity is known to increase the risk of postoperative complications [51]; studies in Asian populations have shown no significant differences in short-term clinical outcomes, suggesting a lack of consensus [52]. Reports on the relationship between obesity and TKA are prevalent in Western literature, but are scarce in Asia, necessitating further examination before using obesity as a predictive factor in Japan.

Sattler et al. [15], conducted a systematic review and meta-analysis identifying predictors for the need for inpatient rehabilitation after TKA, which included age, obesity, comorbidities, and gender as predictive factors. This study found similar factors, reflecting those results. Additionally, although excluded from this study, tools like the Risk Assessment and Prediction Tool, which predicts using multiple factors, have been validated in various countries [13,53], including questions about age and gender, indicating that multiple patient-specific factors are interrelated.

In Japan, factors related to the prediction of hospital stay duration and independent walking have been examined, including the timing of cane walking initiation and physical function [54-55]. However, studies reporting factors related to discharge destinations are scarce, and it is unclear if appropriate destinations are being selected. In cases of extended stays over two weeks, like in Japan, factors other than patient-specific ones, such as postoperative physical function recovery and higher independence in daily and instrumental activities (outdoor walking, stair climbing), are considered.

Limitation

The limitations of this review are threefold. First, the majority of the studies selected are from the USA, with other regions such as Europe under-represented. In Europe, many studies consider joint replacement in general, making it difficult to isolate factors specific to TKA. In addition, there are few reports on preoperative factors associated with discharge destination in Japan, with a limited number of studies and participants for comparison. Second, the effects of surgical factors and osteoarthritis severity, which are associated with postoperative recovery of physical function and discharge destination [56], have not been investigated. Third, in Japan, acute hospital admissions may be influenced by social factors, meaning that patient-specific factors and functionality alone do not determine the length of hospital stay, suggesting that factors beyond those studied may also influence outcomes. To further explore these limitations, it would be necessary to examine factors including social factors preoperatively.

## Conclusions

This study suggests that advanced age, female gender, comorbidities, and obesity are associated with the discharge destinations of patients following TKA. Predicting postoperative outcomes from preoperative factors can facilitate the selection of appropriate discharge destinations, thereby enabling efficient utilization of healthcare resources. Future research should consider preoperative factors in light of Japan's healthcare system, duration of hospital stays, as well as racial and social backgrounds.
